# The Smoking Habit

**Published:** 1919-05-24

**Authors:** 


### The Smoking Habit.

In the *Long Island Medical Journal*, April 19, Dr. Chalmers Jameson presents the following conclusions in connection with the use of tobacco: (1) It must be regarded as a slow poison if used in any considerable amount, and the general opinion that it is always injurious to the young is correct, as it is a poison to the young immature cells; (2) that there are marked differences in susceptibility, and the individual should weigh his own idiosyncrasies as a guide to its use; (3) that the existent physical well-being in the early life of a smoker is not always an index to harmlessness, as degenerative symptoms may and do appear after many years of toleration; (4) that probably what is generally regarded as moderate smoking—the equivalent of five or six cigars a day—borders on immoderation from the standpoint of physical well-being and longevity; (5) that in many instances the balance of good outweighs the bad influence on body and mind; (6) that to render its use comparatively innocuous it should be made less of a continuous habit, and if its use were confined to times of worry, mental stress, and turbulence, and occasional good-fellowship, it would be better for the physical well-being of the world who smoke. Dr. Jameson's remarks impress one as forming a useful review for the consideration by both the practitioner and his patients.

